# Deep Learning-Based Semantic Segmentation and Classification of Otoscopic Images for Otitis Media Diagnosis and Health Promotion

**DOI:** 10.3390/diagnostics16030467

**Published:** 2026-02-02

**Authors:** Chien-Yi Yang, Che-Jui Lee, Wen-Sen Lai, Kuan-Yu Chen, Chung-Feng Kuo, Chieh Hsing Liu, Shao-Cheng Liu

**Affiliations:** 1 Division of General Surgery, Department of Surgery, Tri-Service General Hospital Songshan Branch, National Defense Medical University, Taipei 105309, Taiwan; wayneyoung680324@gmail.com; 2 Department of Health Promotion and Health Education, National Taiwan Normal University, Taipei 106308, Taiwan; 3 Department of Otolaryngology-Head and Neck Surgery, Tri-Service General Hospital, National Defense Medical University, No. 325, Sec. 2, Cheng-Gong Road, Neihu District, Taipei 114202, Taiwan; a5110998@gmail.com; 4 Department of Otolaryngology-Head and Neck Surgery, School of Medicine, College of Medicine, National Defense Medical University, Taipei 114202, Taiwan; lai.vincent99@gmail.com; 5 Department of Otolaryngology-Head and Neck Surgery, Taichung Armed Forces General Hospital, Taichung 411228, Taiwan; 6 Department of Material Science & Engineering, National Taiwan University of Science and Technology, Taipei 106335, Taiwan; ms0709866@gmail.com (K.-Y.C.); jeffreykuo@mail.ntust.edu.tw (C.-F.K.)

**Keywords:** otitis media, image interpretation, computer-assisted, artificial intelligence, neural networks, U-Net

## Abstract

**Background/Objectives:** Otitis media (OM), including acute otitis media (AOM) and chronic otitis media (COM), is a common middle ear disease that can lead to significant morbidity if not accurately diagnosed. Otoscopic interpretation remains subjective and operator-dependent, underscoring the need for objective and reproducible diagnostic support. Recent advances in artificial intelligence (AI) offer promising solutions for automated otoscopic image analysis. **Methods:** We developed an AI-based diagnostic framework consisting of three sequential steps: (1) semi-supervised learning for automatic recognition and semantic segmentation of tympanic membrane structures, (2) region-based feature extraction, and (3) disease classification. A total of 607 clinical otoscopic images were retrospectively collected, including normal ears (*n* = 220), AOM (*n* = 157), and COM with tympanic membrane perforation (*n* = 230). Among these, 485 images were used for training and 122 for independent testing. Semantic segmentation of five anatomically relevant regions was performed using multiple convolutional neural network architectures, including U-Net, PSPNet, HRNet, and DeepLabV3+. Following segmentation, color and texture features were extracted from each region and used to train a neural network-based classifier to differentiate disease states. **Results:** Among the evaluated segmentation models, U-Net demonstrated superior performance, achieving an overall pixel accuracy of 96.76% and a mean Dice similarity coefficient of 71.68%. The segmented regions enabled reliable extraction of discriminative chromatic and texture features. In the final classification stage, the proposed framework achieved diagnostic accuracies of 100% for normal ears, 100% for AOM, and 91.3% for COM on the independent test set, with an overall accuracy of 96.72%. **Conclusions:** This study demonstrates that a semi-supervised, segmentation-driven AI pipeline integrating feature extraction and classification can achieve high diagnostic accuracy for otitis media. The proposed framework offers a clinically interpretable and fully automated approach that may enhance diagnostic consistency, support clinical decision-making, and facilitate scalable otoscopic assessment in diverse healthcare screening settings for disease prevention and health education.

## 1. Introduction

Otitis media (OM) represents a heterogeneous group of infectious and inflammatory diseases affecting the middle ear and remains one of the most common conditions encountered in otolaryngology practice [1]. Clinically, OM is broadly classified into acute otitis media (AOM) and chronic otitis media (COM), each with distinct pathophysiology, clinical presentation, and management strategies. AOM is an acute inflammatory condition, most frequently caused by bacterial pathogens. The diagnosis of AOM relies on a combination of clinical symptoms—such as acute ear pain, fever, and irritability—and otoscopic findings demonstrating middle ear effusion with signs of acute inflammation [2]. Management primarily focuses on pain control and appropriate antibiotic therapy. In contrast, COM is characterized by persistent or recurrent inflammation of the middle ear, often associated with chronic otorrhea through a perforated tympanic membrane. Diagnosis is usually established through otoscopic identification of tympanic membrane perforation and ongoing purulent discharge. Treatment strategies for COM commonly involve topical antibiotic therapy to control infection and achieve a dry ear, followed by surgical repair. Tympanoplasty is frequently performed to restore tympanic membrane integrity, with reported closure rates of approximately 83–87% [3]. In cases complicated by cholesteatoma, surgical removal is mandatory, as it remains the only definitive treatment and is essential for preventing progressive bone erosion and intracranial complications [4].

Although otoscopy is the cornerstone of OM diagnosis, its interpretation is inherently subjective and highly dependent on clinician experience and training. Variability in diagnostic accuracy, limited access to specialist expertise, and the increasing concern of antibiotic overuse and resistance highlight the need for more objective, standardized, and reproducible diagnostic tools [5]. In recent years, deep neural networks and other machine learning (ML) approaches have demonstrated considerable potential in the automated analysis of medical images, including the detection of tympanic membrane abnormalities from otoscopic images [6,7]. Among these approaches, convolutional neural networks (CNNs) have shown particular effectiveness due to their ability to learn complex spatial and textural features directly from visual data. The integration of artificial intelligence (AI)-driven semantic segmentation into otologic imaging analysis offers a promising avenue for improving diagnostic accuracy, consistency, and efficiency in clinical practice [8]. However, most previously published studies have relied on manually preselected otoscopic images that explicitly contain the tympanic membrane. This human-dependent region-of-interest (ROI) selection process inevitably introduces selection bias and limits both the reproducibility of AI training and the generalizability of the resulting models. In real-world clinical practice, otoscopic images frequently do not adequately visualize the tympanic membrane or are contaminated by cerumen or suboptimal imaging angles, which poses a significant challenge for practical AI deployment. In contrast, the proposed framework is capable of automatically determining whether a valid tympanic membrane is present within an input image. This design feature enhances robustness in real-world clinical settings and represents a critical step toward the practical implementation of AI-assisted otoscopic diagnostics.

The primary objective of this study is threefold. First, we aim to evaluate and compare the performance of four state-of-the-art semantic segmentation architectures—PSPNet, HRNet, DeepLabV3+ with a ResNet backbone, and U-Net—in the automatic detection and precise segmentation of tympanic membrane structures from clinical otoscopic images. The accuracy of these models will be assessed by their ability to correctly identify regions of interest (ROIs) relative to non-ROI areas, thereby determining the most reliable architecture for tympanic membrane segmentation. Second, following automated segmentation, we will perform feature extraction using deep learning-based and conventional ML techniques applied to the segmented anatomical regions. By analyzing color, texture, and structural features within predefined tympanic membrane subregions, the proposed framework aims to distinguish between normal ears, AOM, and COM with tympanic membrane perforation requiring surgical intervention. This region-based analytical strategy is designed to more closely reflect clinical reasoning and to enhance diagnostic interpretability. Finally, in light of the heightened risks associated with in-person clinical evaluations during the COVID-19 pandemic and potential future outbreaks of respiratory infectious diseases, this study seeks to address the growing demand for non-invasive, remote, and automated diagnostic tools. By leveraging AI-assisted analysis of otoscopic images, the proposed system has the potential to support clinical decision-making, facilitate early and accurate diagnosis, reduce unnecessary antibiotic use, and ultimately improve patient outcomes and promote health in both routine and resource-limited healthcare settings.

## 2. Materials and Methods

### 2.1. Data Procurement and Semi-Supervised Learning

**Figure 1:**
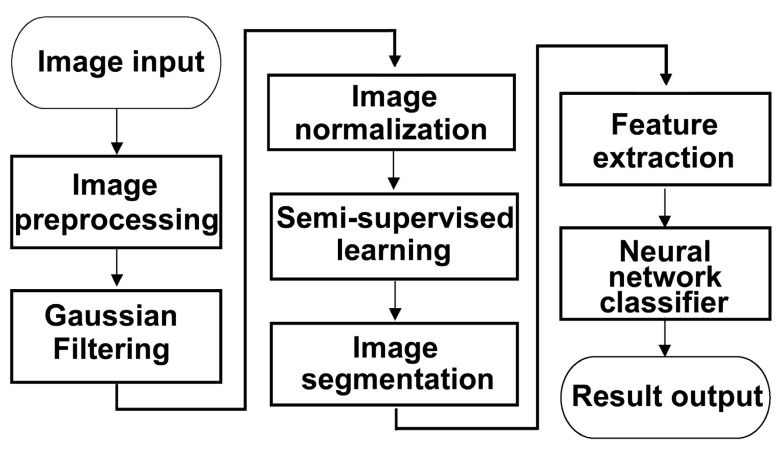
Overview of the image processing and artificial intelligence (AI) training workflow for otoscopic image analysis.

This study performed AI-based image analysis using a dataset composed exclusively of clinical otoscopic images. From January 2020 to December 2021, otoscopic images were retrospectively collected from 607 patients aged 20–80 years. The dataset comprised 220 normal otoscopic images, 157 images of AOM, and 230 images of COM with tympanic membrane perforation requiring surgical intervention. Among the total dataset, 485 images (79.9%) were manually annotated and used for model training. Semantic segmentation was performed on five anatomically relevant regions: the external auditory canal, tympanic ring, pars tensa, pars flaccida, and handle of the malleus. All regions were carefully delineated and labeled by a board-certified otolaryngologist. To ensure annotation accuracy and consistency, each segmentation mask underwent independent verification by three additional otolaryngologists, thereby establishing a standardized and reliable ground truth. The remaining 122 images (20.1%) were withheld for validation and testing, enabling objective performance evaluation on unseen data. In addition to images containing clearly visible tympanic membranes, a subset of images intentionally lacking an identifiable tympanic membrane was also collected from the aforementioned 220 normal cases. These images were designated as “invalid otoscopic images” and were deliberately included in the dataset to train the model to distinguish between diagnostically valid and invalid images, thereby improving its robustness and real-world applicability in clinical settings. To further evaluate model generalizability, external validation was performed using an independent dataset collected from another hospital, comprising 52 cases acquired with a different endoscopic imaging system. The overall image processing workflow is summarized in Figure 1.

To maximize the utility of both labeled and unlabeled data, a semi-supervised learning strategy incorporating pseudo-labeling was adopted. Initially, a CNN was trained using the manually labeled images to establish a baseline model. This model was then applied to unlabeled images, where class probabilities were estimated by minimizing entropy to reduce uncertainty and class overlap. The class with the highest predicted probability was assigned as a pseudo-label and incorporated into subsequent training iterations. To mitigate error propagation, only predictions exceeding a predefined confidence threshold were retained, ensuring that low-confidence pseudo-labels did not degrade model performance. In parallel, a loss-based mixture model was employed to dynamically separate clean, confidently labeled samples from noisy or uncertain data during training. This adaptive framework enabled the model to learn from the full dataset in a semi-supervised manner, thereby enhancing robustness and generalizability. Feature parameters extracted from the segmented regions were subsequently compiled into structured datasets and analyzed using multiple predictive models, including logistic regression, decision trees, k-nearest neighbors (KNN), and ensemble learning methods, to improve diagnostic accuracy and alignment with clinical outcomes. Several state-of-the-art semantic segmentation models, including U-Net, PSPNet, HRNet, and DeepLabV3+ with a ResNet backbone, were implemented and systematically compared. Based on quantitative segmentation accuracy, the best-performing model was selected and used for subsequent feature extraction and diagnostic classification.

### 2.2. Color and Texture Feature Extraction

Following semantic segmentation, chromatic and texture features were extracted from the 485 annotated otoscopic images, including 176 normal cases, 125 AOM cases, and 184 COM cases. Feature extraction was performed independently for each of the five segmented anatomical regions. Color analysis focused on six chromatic parameters: Red (R), Green (G), Blue (B), Hue (H), Chroma Blue (Cb), and Chroma Red (Cr). These parameters were selected to capture disease-related color variations across the tympanic membrane and surrounding structures. Statistical analyses were conducted on each region to characterize condition-specific chromatic patterns. Texture features were extracted using the Gray-Level Co-occurrence Matrix (GLCM), which quantifies spatial relationships between pixel gray levels and is well suited for assessing structural changes associated with inflammation. Haralick texture features were derived from the GLCM, including Angular Second Moment (ASM), Contrast (CON), Correlation (COR), Variance, Inverse Difference Moment (IDM/Homogeneity), Sum Average, Sum Variance, Sum Entropy, Energy (ENG), Difference Variance, Difference Entropy, Information Measure of Correlation 1 (IMC1), Information Measure of Correlation 2 (IMC2), and Maximal Correlation Coefficient (MCC). These texture descriptors provided complementary structural information that enhanced discrimination among normal ears, AOM, and COM.

### 2.3. Image Classification and Performance Evaluation

**Figure 2:**
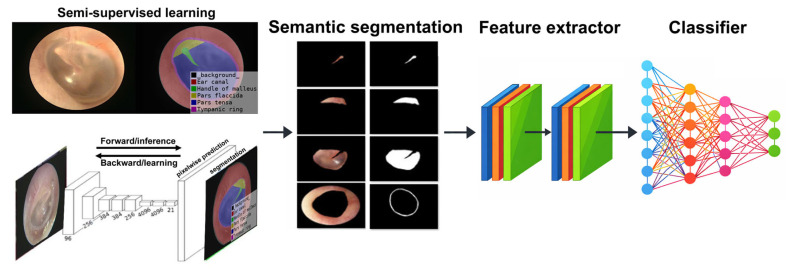
Study pipeline for quantitative otoscopic image analysis. The proposed framework integrates semi-supervised learning for anatomical annotation, followed by semantic segmentation, region-based feature extraction, and automated image classification.

The complete system pipeline begins with semantic segmentation of otoscopic images to identify the five regions of interest within the tympanic membrane. Color and texture features are then extracted from each region and used as inputs for image classification. As part of the proposed framework, the system was intentionally designed to first determine whether an input image constituted a clinically meaningful otoscopic image, defined by the presence of recognizable ear anatomy. Images that failed this initial validity assessment were automatically excluded at the first stage of analysis. For images that passed the validity check, a CNN-based classifier was developed to categorize images into three diagnostic classes: normal, AOM, and COM (Figure 2). Owing to the prior segmentation and feature extraction steps, the classification process required minimal manual relabeling. Instead, supervised learning focused on refining class boundaries using region-specific chromatic and texture information. Within the CNN architecture, activation functions served as selective filters, allowing neuronal activation only when predefined thresholds were exceeded. This mechanism enhanced the model’s ability to identify clinically relevant patterns. The classifier integrated comprehensive hue distributions with GLCM-derived texture metrics, enabling effective characterization of both color and structural abnormalities.

Final model performance was evaluated using the 122 reserved test images. A confusion matrix was constructed to quantify true positives, true negatives, false positives, and false negatives across all diagnostic categories. The accuracy was defined as (TP + TN)/(TP + TN + FP + FN), while the recall was defined as TP/(TP + FN) and the Specificity was defined as TN/(TN + FP). This analysis provided a transparent assessment of classification accuracy, robustness, and potential areas for further optimization.

### 2.4. Ethical Considerations

The research protocol (NO: 1-108-05-132) was reviewed and approved by the Institutional Review Board.

## 3. Result

### 3.1. Tympanic Membrane Segmentation

**Figure 3:**
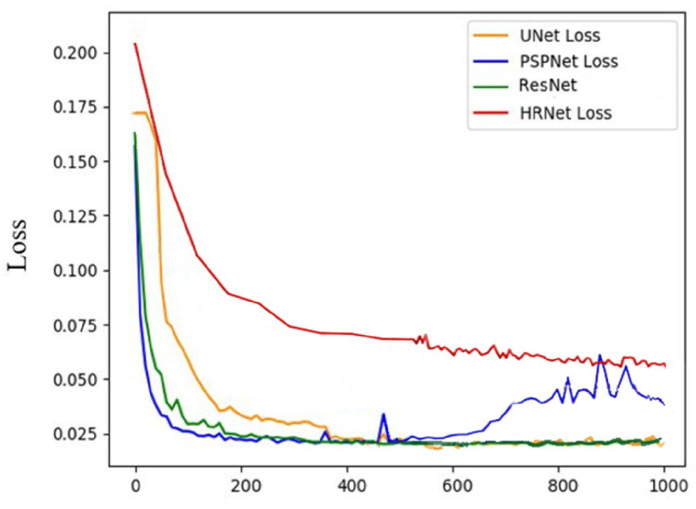
Validation loss curves of different semantic segmentation models, including U-Net, PSPNet, HRNet, and DeepLabV3+. The decreasing loss values indicate progressive model learning and improved segmentation performance during training.

**Figure 4:**
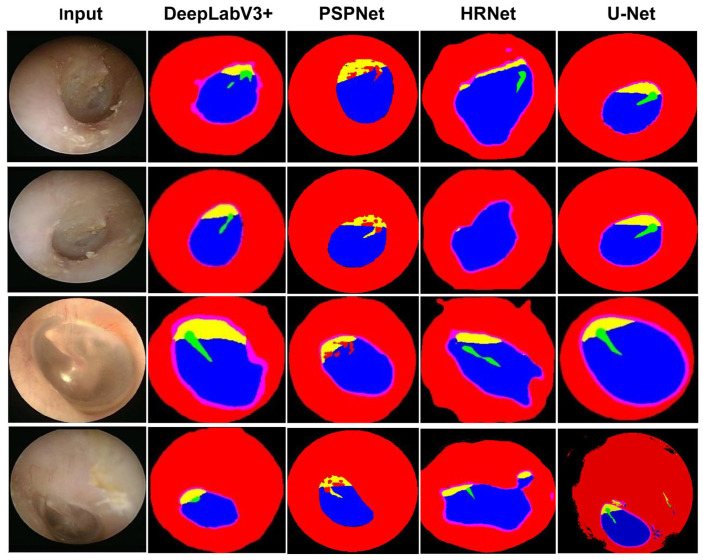
Comparison of segmentation results obtained using the state-of-the-art networks. Each row represents a different otoscopic input image. From left to right, columns show the original image and segmentation outputs generated by DeepLabV3+, PSPNet, HRNet, and U-Net, respectively. Color-coded regions correspond to anatomical structures: pars flaccida (yellow), pars tensa (blue), tympanic ring (purple), handle of the malleus (green), and ear canal (red).

The overall workflow for image processing and AI model training is illustrated in Figure 1. Semantic segmentation performance was evaluated using multiple deep learning architectures, with ground truth established by consensus among three board-certified otolaryngologists. A total of 485 otoscopic images were used for training and validation. Among the evaluated models, U-Net demonstrated superior segmentation performance, achieving the highest Pixel Accuracy (PA) and Dice Similarity Coefficient (DICE) across all anatomical regions (Table 1) (Figure 3). Consequently, U-Net was selected for subsequent analyses. Visual inspection further confirmed accurate delineation of the five anatomical structures—external auditory canal, tympanic ring, pars tensa, pars flaccida, and handle of the malleus—as illustrated in Figure 4. Quantitative comparison between U-Net-based segmentation and expert annotations revealed high region-specific accuracy. The segmentation accuracy reached 99.94% for the external auditory canal, 98.53% for the tympanic ring, 95.67% for the pars tensa, 95.43% for the pars flaccida, and 97.57% for the handle of the malleus, resulting in an overall segmentation accuracy of 96.76%.

### 3.2. Feature Learning and Disease Classification

Following semantic segmentation, five anatomically defined regions were automatically identified in each otoscopic image (Figure 2). Feature extraction was subsequently performed on all 485 images, including normal ears, AOM, and COM with tympanic membrane perforation. From each segmented region, six color features and four texture features were extracted. Color features included RGB channels, hue from the HSV color space, and chromatic components, while texture analysis was conducted using the GLCM. In total, 28 feature parameters were compiled and used as input variables for the artificial neural network (ANN) classifier. Different feature combinations were systematically evaluated by training and validating an ANN classifier. Classification accuracy, confusion matrix performance, and model convergence stability were jointly considered as objective evaluation criteria. Through multiple experimental comparisons, we observed that certain features contributed limited discriminative power, increased model complexity, or introduced redundancy that adversely affected convergence stability. These features were excluded and this process represents a performance-driven, wrapper-style feature selection approach to classification. Ultimately, the eight most informative features were selected because they consistently demonstrated stable performance and stronger discriminative capability for otitis media classification.

**Figure 5:**
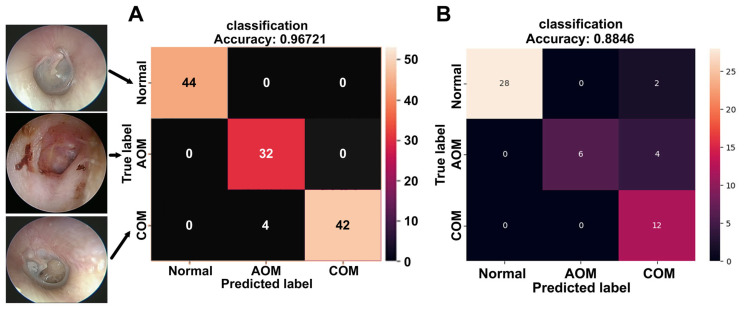
Confusion matrices illustrating the classification performance of the proposed model for normal tympanic membranes, acute otitis media (AOM), and chronic otitis media (COM) with tympanic membrane perforation. (**A**) In the internal dataset, the model achieved accuracies of 100% for normal cases, 100% for AOM, and 91.3% for COM, with an overall accuracy of 96.72%. (**B**) In the external validation cohort, the overall accuracy was 88.46%, with a sensitivity of 88.46%, specificity of 93.33%, precision of 90.00%, and an F-score of 0.8571.

Under optimized training conditions, the ANN achieved a peak classification accuracy of 94.14%, with a corresponding loss value of 0.0586 and validation accuracy of 96.88%. For final diagnostic evaluation, the model was trained using 485 images and tested on an independent set of 122 images. The confusion matrix-based heatmap demonstrated excellent diagnostic performance (Figure 5A). All 44 images predicted as normal were correctly classified (100% accuracy), as were all 32 images predicted as AOM (100% accuracy). Among the 46 images predicted as COM, 42 were correctly classified, resulting in an accuracy of 91.3%. The overall diagnostic accuracy across all categories reached 96.72%, with a sensitivity of 94.87%, specificity of 100%, precision of 100%, and an F-score of 0.9736. In addition, 52 cases from a different hospital, acquired using a distinct endoscopic imaging system, were included for external validation. In this external cohort, the proposed system achieved an overall accuracy of 88.46% (46/52), with a sensitivity of 88.46%, specificity of 93.33%, precision of 90.00%, and an F-score of 0.8571 (Figure 5B).

## 4. Discussion

Otoscopic examination remains a cornerstone in the diagnosis of middle ear diseases, particularly otitis media. However, accurate interpretation of otoscopic findings requires substantial clinical experience, and diagnostic variability persists even among trained otolaryngologists [9]. With the increasing adoption of video otoscopy in clinical practice, large volumes of high-quality image data have become available, creating new opportunities for machine learning-based image analysis [10,11]. AI-assisted interpretation of otoscopic images has the potential to support clinical decision-making, especially for less experienced physicians or in resource-limited settings. Semantic segmentation plays a critical role in medical image analysis by enabling precise localization of anatomically and clinically relevant regions of interest (ROIs) [12]. In otoscopic imaging, segmentation of the tympanic membrane and adjacent structures is particularly challenging due to anatomical variability, inconsistent illumination, cerumen obstruction, and inflammatory changes. In the present study, we applied a deep learning-based semantic segmentation framework to delineate five key anatomical regions from clinical otoscopic images. By focusing on anatomically meaningful ROIs rather than relying solely on global image-level classification, our approach enables region-specific feature extraction that more closely mirrors clinical reasoning during otoscopic assessment.

Several previous studies have explored AI-based approaches for the segmentation and classification of otoscopic images [13]. Shie et al. employed an active contour method to segment the tympanic membrane and extracted handcrafted features, including a gray-level co-occurrence matrix (GLCM), histogram of oriented gradients (HOG), local binary patterns (LBPs), and Gabor features, achieving a classification accuracy of 88.06% using an AdaBoost classifier [14]. Pham et al. proposed EAR-UNet, incorporating EfficientNet and attention mechanisms, and reported a segmentation accuracy of 95.8% with favorable Dice similarity coefficients [15]. Kim et al. integrated ResNet152 into a UNet++ architecture to further enhance segmentation performance using an augmented dataset [16]. Other studies have utilized fully convolutional networks with hybrid loss functions combining Dice and active contour losses to improve boundary delineation. Collectively, these studies highlight the rapid evolution of deep learning techniques in otoscopic image analysis and the importance of accurate segmentation for downstream diagnostic tasks [17]. However, a critical limitation of most previous approaches is their reliance on manually pre-selected images that are confirmed to contain a visible tympanic membrane [18]. Such manual ROI validation introduces selection bias and limits reproducibility and scalability in real-world clinical applications. In contrast, a key strength of the present study is the development of a fully automated pipeline capable of determining whether an input image contains a valid tympanic membrane. Images lacking an identifiable eardrum or compromised by severe contamination (e.g., ear discharge), which are common in routine clinical examinations, are automatically recognized as invalid and excluded from further analysis. This design more closely reflects real-world clinical workflows and enhances the robustness and generalizability of the proposed system.

A recent review of AI applications in middle ear disease diagnosis categorized existing studies into classification-only, segmentation-only, and combined segmentation–classification approaches [19,20]. While classification-only models demonstrated an average accuracy of approximately 86%, studies integrating both segmentation and classification achieved higher average accuracies of around 90.8%. In this study, our model demonstrated high accuracy across multiple clinically relevant metrics. For COM, the system achieved an accuracy of 91.3%, with high sensitivity and specificity, and an F-score exceeding 0.97, indicating balanced performance between precision and recall. Importantly, these results extend beyond overall accuracy alone and address reviewer concerns regarding clinically meaningful performance indicators. To further assess generalizability, we conducted external validation using data collected from a different hospital with a distinct endoscopic imaging system. Despite differences in imaging settings, the model maintained favorable diagnostic performance, with an overall accuracy of 88.46%, sensitivity of 88.46%, specificity of 93.33%, and an F-score of 0.8571. These findings support the robustness of the proposed approach under heterogeneous real-world conditions. By integrating U-Net–based semantic segmentation with region-specific color and texture feature extraction, our model captures both visual appearance and structural alterations associated with acute and chronic otitis media. The use of chromatic parameters combined with GLCM-derived texture features allows for interpretable feature representation and aligns well with how clinicians evaluate otoscopic findings. Compared with classification-only approaches, this segmentation-guided strategy provides a more transparent and clinically intuitive framework for automated otoscopic diagnosis.

Beyond diagnostic accuracy, AI-assisted image segmentation offers broader clinical implications. Precise identification of anatomical structures may facilitate longitudinal disease monitoring, treatment response assessment, and preoperative planning, particularly in patients with chronic otitis media requiring surgical intervention. Moreover, by filtering out non-diagnostic images at the outset, the system may reduce diagnostic errors, minimize unnecessary antibiotic prescriptions, and support more appropriate referral and triage decisions. Improved diagnostic precision may reduce unnecessary antibiotic prescriptions and support more appropriate referral decisions [21,22]. Nevertheless, successful clinical deployment of AI systems requires careful consideration of real-world challenges, including image quality variability, differences in endoscopic equipment, and regulatory and ethical considerations [23].

Several limitations of this study should be acknowledged. First, model performance is inherently dependent on the quality and diversity of the training dataset. Although our dataset included normal ears, AOM, and COM with tympanic membrane perforation, other disease subtypes and intermediate stages were not fully represented. Second, variations in endoscopic devices, illumination, and patient anatomy may affect segmentation robustness when applied to external datasets. Finally, this was a retrospective, single-institution study, which may limit generalizability despite the inclusion of external validation data. Future work should incorporate multi-center data, broader disease spectra, and prospective validation to further strengthen clinical applicability. Despite these limitations, this study demonstrates that deep learning-based semantic segmentation combined with automated image validity assessment and region-specific feature extraction can achieve high and clinically meaningful diagnostic performance in otoscopic image analysis. Such AI-assisted systems have the potential to reduce diagnostic variability, alleviate clinical workload, and support more efficient and objective diagnosis and management of middle ear diseases. Moreover, by improving accessibility to objective otoscopic assessments and enhancing disease awareness, these technologies may encourage earlier health-seeking behaviors and support longitudinal, personalized disease management strategies [24]. Collectively, our findings support the role of AI-based tools as adjunctive systems in otitis media diagnosis and underscore their promise in enhancing diagnostic consistency and clinical decision-making in otolaryngology.

## 5. Conclusions

Our research highlights the potential of using U-Net for fully automated semantic segmentation of tympanic membrane structures. This ability supports subsequent feature extraction and machine learning, allowing for accurate otitis media diagnosis. By successfully differentiating between normal tympanic membranes, AOM, and COM, our study enhances automated medical assessments and improves healthcare service efficiency. This innovation is poised to increase the accuracy and speed of otitis media evaluations, providing benefits to both healthcare providers and patients. By enabling the public to fully understand their disease exposure through AI, it can foster disease awareness, thereby increasing their willingness to promote health and triggering health-promoting behaviors.

## Figures and Tables

**Table 1:** Diagnostic performance of different semantic segmentation models. Among the evaluated architectures, U-Net demonstrated superior performance across all assessed metrics. PA, pixel accuracy; mIoU, mean Intersection over Union.

Model	PA	Dice Coefficient	mIoU
PSPNet	77.31%	75.25%	82.16%
HRNet	81.32%	76.88%	79.02%
DeepLabV3+	95.55%	71.31%	83.69%
U-Net	96.76%	71.68%	82.63%

## Data Availability

The raw data supporting the conclusions of this article will be made available by the authors on request.
